# Comprehensive Analysis of Bacterial Communities and Microbiological Quality of Frozen Edible Insects

**DOI:** 10.3390/foods14132347

**Published:** 2025-07-01

**Authors:** Sasiprapa Krongdang, Nipitpong Sawongta, Jintana Pheepakpraw, Achirawit Ngamsomchat, Sutee Wangtueai, Jittimon Wongsa, Thanya Parametthanuwat, Narin Charoenphun, Thararat Chitov

**Affiliations:** 1 Faculty of Science and Social Sciences, Burapha University, Sakaeo Campus, Sakaeo 27160, Thailand; sasiprapa.kr@buu.ac.th; 2 Department of Biology, Faculty of Science, Chiang Mai University, Chiang Mai 50200, Thailand; nipitpong_s@cmu.ac.th (N.S.); jintana_ph@cmu.ac.th (J.P.); 3 Office of Research Administration, Chiang Mai University, Chiang Mai 50200, Thailand; achirawit.ng@cmu.ac.th; 4 School of Agro-Industry, Faculty of Agro-Industry, Chiang Mai University, Chiang Mai 50100, Thailand; sutee.w@cmu.ac.th; 5 Faculty of Industrial Technology and Management, King Mongkut’s University of Technology North Bangkok, Prachinburi Campus, Prachinburi 25230, Thailand; jittimon.w@itm.kmutnb.ac.th (J.W.); thanya.p@itm.kmutnb.ac.th (T.P.); 6 Food and Agro-Industry Research Center, King Mongkut’s University of Technology North Bangkok, Bangkok 10800, Thailand; 7 KMUTNB Techno Park Prachinburi, King Mongkut’s University of Technology North Bangkok, Prachinburi Campus, Muang, Prachinburi 25230, Thailand; 8 Faculty of Science and Arts, Burapha University, Chanthaburi Campus, Chanthaburi 22170, Thailand; 9 Environmental Science Research Center (ESRC), Faculty of Science, Chiang Mai University, Chiang Mai 50200, Thailand

**Keywords:** entomophagy, foodborne pathogens, metagenomics, microbiological risk assessment, food safety, sustainable food source, novel food

## Abstract

Edible insects are gaining traction worldwide; however, the existing data regarding their microbiological quality remain inadequate. This study investigated the bacterial communities and microbiological quality of five types of frozen edible insects commercially available in Thailand. Amplicon sequencing revealed Firmicutes (Bacillota) and Proteobacteria (Pseudomonadota) as the main phyla across all samples; Bacteroidota was predominant in house crickets, Actinobacteriota in silkworms, and Desulfobacterota was exclusively found in house and mole crickets. Culture-based assays showed total viable counts, lactic acid bacteria, yeasts–molds, and spore-formers ranging from 3.41–6.58, 2.52–7.41, 1.83–5.62, to 2.00–4.70 log CFU·g^−1^, respectively. In some samples, *Enterobacteriaceae* and *Escherichia coli*, key hygiene indicators, reached 5.05 and 2.70 log CFU·g^−1^, respectively. Among foodborne pathogens, presumptive *Bacillus cereus* was found to vary from <1.70 to 3.93 log CFU·g^−1^, while *Clostridium perfringens* and *Staphylococcus aureus* were under the quantitation limit, and *Salmonella* was absent. Overall, the results indicate significant variation in microbial diversity and quality among different insect types. The high levels of microbial hygiene indicators and foodborne pathogens in some samples raised food safety concerns and point to the need to develop or implement production guidelines and microbiological criteria for frozen edible insects to ensure food safety.

## 1. Introduction

Entomophagy, or the consumption of insects as a food source, has a historical and cultural practice spanning many thousands of years [1,2]. Edible insects are abundant in proteins, essential amino acids, fats, and micronutrients, and they possess various functional properties [3,4]. Edible insect rearing exhibits a significantly lower environmental impact relative to conventional livestock production, thereby aligning with the objectives of the Sustainable Development Goals (SDGs) [5,6,7]. It promotes sustainable consumption and production, making use of minimum resources, and can reduce a negative impact of climate change by reducing emissions of greenhouse gases (methane, nitrous oxide, and carbon dioxide) directly or indirectly produced through cattle farming, thus specifically promoting the achievement of SDGs 12 and 13 [7].

The recognition of edible insects as a sustainable and nutritious food source has significantly grown in recent years. However, this increasing acknowledgment is accompanied by persistent concerns regarding their safety for human consumption [8,9]. Edible insects can have several potential health risks from allergens and contamination of chemical and microbial pathogens hazards [2]. These safety concerns have become particularly intriguing in the risk assessment of edible insects and insect-based products among consumers, food manufacturers, and food authorities [10].

In Europe, edible insects and insect-based products are classified as novel foods according to Regulation (EU) 2015/2283 and its Commission Implementing Regulations 2017/2468 and 2017/2469 [11]. These regulations require that safety assessment and authorization be imposed by the European Commission (EC), and all novel foods must undergo a risk assessment conducted by the European Food Safety Authority (EFSA) [10]. Based on EFSA’s assessments, the commission has approved the farming and processing of specific insect species [12]. This framework ensures that only insect species passing rigorous safety evaluations are allowed in the EU market, balancing innovation with consumer safety in this emerging food sector. In Thailand, criteria have been established for certain economically significant edible insects, reflecting the rapid growth of the edible insect market and industries in recent years [13]. An example is the guidance provided by the Food and Agriculture Organization (FAO) regarding sustainable cricket farming [14]. The establishment of microbiological criteria is challenging due to its dependence on available data. Studies have evaluated the microbiological quality of edible insects using metagenomic/metagenetic and culturing methods, revealing a diverse array of bacterial genera [15,16]. Some research has identified and characterized microbiological hazards, including *Staphylococcus aureus* and *Salmonella* [17]. Data regarding the microbiological quality of edible insects, however, remain limited, attributed to the extensive variety of species, lack of standardized rearing and processing methods, and the diverse geographical contexts.

This study, therefore, aimed to investigate the diversity of bacterial communities in various types of frozen insects using metagenetic analysis and assessing microbiological quality, including potential microbiological hazards, through culturing methods. This study focused on edible insect types that are either industrially produced or commercially available, with potential for global consumption. The anticipated outcomes aim to expand the database concerning the microbiological qualities of frozen edible insects and to offer guidance for processing protocols and the establishment of microbiological criteria to ensure food safety.

## 2. Materials and Methods

### 2.1. Sample Collection

**Figure 1:**
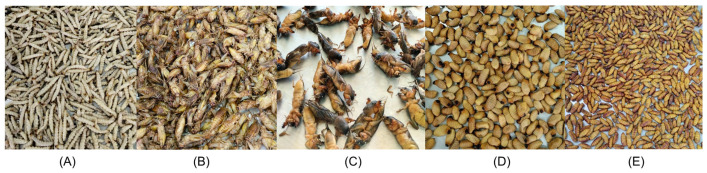
Frozen edible insects used in this study. (**A**) Bamboo worms or bamboo borers (*Omphisa fuscidentalis*), (**B**) house crickets (*Acheta domestica*), (**C**) mole crickets (*Gryllotalpa orientalis*), (**D**) red palm weevil larvae (*Rhynchophorus ferrugineus*), and (**E**) silkworms or silk moth larvae (*Bombyx mori*).

Five types of frozen edible insects used in this study included bamboo worms or bamboo borers (BW) (*Omphisa fuscidentalis* H.), house crickets (HC) (*Acheta domesticus*), mole crickets (MC) (*Gryllotalpa orientalis*), red palm weevil larvae (PW) (*Rhynchophorus ferrugineus*), and silkworms or silk moth larvae (SM) (*Bombyx mori* L.) (Figure 1). Frozen insects were acquired from various retailers during February and March 2024. The experiment was designed to include five varieties of edible insects, employing six biological replicates (*n* = 6; total sample size = 30).

### 2.2. DNA Extraction

A 30 g sample of each frozen insect was ground into a fine powder with the aid of liquid nitrogen. A 5 g sample was placed in a 100 mm mesh nylon filter bag, which was subsequently placed in a 50 mL conical tube, followed by the addition of 25 mL of Butterfield’s phosphate buffer. The tube’s contents were mixed with a vortex mixer and centrifuged at 7000 rpm for 20 min, after which the supernatant was discarded. The pellet was utilized for DNA extraction through a method that employs cetyltrimethylammonium bromide (CTAB) [18]. The cell pellet was treated with CTAB buffer, proteinase K, and SDS, followed by incubation at 60 °C for 1 h. The supernatant was extracted using chloroform: isoamyl alcohol (24:1). The aqueous phase was then transferred to a new microtube, and DNA was precipitated with isopropanol. The DNA pellet, obtained post-centrifugation, underwent two washes with 70% ethanol, was air-dried, and was subsequently resuspended in TE buffer. The DNA samples were maintained at −20 °C.

### 2.3. 16S rRNA Gene Amplicon Sequencing

The extracted DNA was used to study bacterial diversity by targeting hypervariable regions V3 and V4 of the 16S rRNA gene, employing 341F/805R primers [19]. Targeted amplicon libraries were produced and sequenced utilizing an Illumina NovaSeq 6000 platform (Illumina, Inc., San Diego, CA, USA) in paired-end mode, with sequencing conducted by U2Bio in Seoul, Korea. All sequences can be accessed in the Sequence Read Archive (SRA) at the National Center for Biotechnology Information (NCBI) using accession number PRJNA1257548.

### 2.4. Microbiome Sequencing and Analysis

DADA2 was employed to process raw reads, including denoising, filtering, merging, and chimera removal, to generate amplicon sequence variants (ASVs) [20]. Representative sequences for each ASV, following the chimera removal process, were taxonomically assigned using the SILVA v.138.2 reference training dataset. The “assignTaxonomy” function was employed to achieve taxonomic assignment at the species level through exact matching with the SILVA v.138.2 dataset (https://www.arb-silva.de/ (accessed on 22 January 2025). Microbiome profiles were generated using the phyloseq package in R version 4.4.3. Relative abundance was utilized to depict taxonomic profiles at the phylum, family, and genus levels. The relative abundance of ASVs with less than 1% was categorized as “others.” The total microbial relative abundance variation across the samples was evaluated using the Kruskal-Wallis test, followed by Dunn’s post hoc tests. Alpha diversity was assessed with the “vegan” package to generate Observed ASVs, Chao1, Shannon, and Simpson indices. The differences in alpha diversity among groups were analyzed using the Kruskal-Wallis test, followed by the Wilcoxon test for pairwise comparisons. Beta diversity was assessed through non-metric multidimensional scaling (NMDS), principal coordinate analysis (PCoA) utilizing Bray-Curtis dissimilarity metrics, and weighted UniFrac phylogenetic distances. Group differences were assessed using PERMANOVA models (Adonis) to analyze the impact of insect groups on combined beta diversity, along with an analysis of similarities (ANOSIM), and visualized through NMDS and PCoA plots. Functional pathways were predicted and mapped (MetaCyc pathways) using the ENZYME nomenclature database in conjunction with PICRUSt2 software version 2.5.3 [21]. A heatmap was created to illustrate the hierarchical clustering of each predicted gene. The Linear Discriminant Analysis Effect Size (LEfSe) method, employing an LDA effect size threshold greater than 2.0, was utilized alongside the Kruskal-Wallis test (*p* ≤ 0.01) to identify and differentiate pathways [22].

### 2.5. Microbiological Analysis of Frozen Edible Insects

#### 2.5.1. Sample Preparation

All frozen insect samples of each type were rapidly thawed at room temperature. Each sample of whole insects was cut into small pieces with sterile scissors. A 50 g portion of the insect sample was combined with 450 mL of Butterfield’s phosphate-buffered dilution water (0.0425 g/L KH_2_PO_4_ in water) in a stomacher bag. The mixture was homogenized with a stomacher (Seward, Worthing, UK) at high speed for 2 min. Subsequent ten-fold serial dilutions were prepared to attain the required dilution levels for microbial enumeration.

#### 2.5.2. Enumeration of Microbial Flora

The microbial flora, comprising total bacteria quantified as total viable count (TVC), lactic acid bacteria (LAB), yeasts and molds (YM), and spore-forming bacteria (SFB), were quantitatively analyzed for all types of edible insects, with six samples per type. Initially, ten-fold dilutions (10^−1^ to 10^−4^) were prepared for the analysis of each sample. The total viable count (TVC) was assessed using the pour plate technique as described in the US FDA’s Bacteriological Analytical Manual (BAM) (Chapter 3) [23], wherein a 1 mL aliquot from each dilution was inoculated in Plate Count Agar using the pour plate technique. The plates were incubated for 48 h at 37 °C. LAB were enumerated using spread plating (0.1 mL from each dilution) on De Man–Rogosa–Sharpe agar (BioMérieux, Marcy L’Etoile, France) supplemented with cysteine and bromophenol blue (MRS-Cys-BPB). The plates were incubated for 72 h at 37 °C in a modified gaseous atmosphere utilizing GENbox anaer gas modifier packs (BioMérieux, Marcy L’Etoile, France) [24]. Yeast and mold counts were assessed using the spread plate method on Dichloran Rose Bengal Chloramphenicol (DRBC) agar (Difco and BBL, Sparks, MD, USA), following the protocol described in the US FDA’s BAM (Chapter 18) [25]. The plates underwent incubation at 25 °C for a duration of 5 days. Samples for SFB were initially heated in a water bath at 80 °C for 10 min to eradicate vegetative cells [26]. Following rapid cooling, ten-fold serial dilutions were performed, and a 0.1 mL aliquot from each dilution (10^−1^ to 10^−2^) was spread onto Tryptic Soy Agar (TSA) (Difco and BBL, MD, USA). The plates underwent incubation for 48 h at 37 °C. Each sample was plated in duplicate. Colony counts were conducted post-incubation for all enumeration methods, and averages were calculated as CFU and log CFU per gram (log CFU/g) of sample.

#### 2.5.3. Analysis of Indicator Microorganisms

The enumeration of *Enterobacteriaceae* was conducted in accordance with ISO 21528-2:2017 [27], utilizing the pour plate method (1 mL, from dilutions 10^−1^ to 10^−3^) with Violet Red Bile Glucose (VRBG) agar (BioMérieux, Marcy L’Etoile, France). Following solidification, 5–10 mL of VRBG agar was overlaid and allowed to solidify. The plates were incubated for 24 h at 37 °C. *Escherichia coli* was quantified using the pour plate method (1 mL, from dilution 10^−1^) on ChromID Coli^®^ agar (BioMérieux, Marcy L’Etoile, France) in accordance with the ISO 16649-2 standard [28]. Each sample was plated in duplicate and incubated for 24 h at 44 °C. Colonies exhibiting a reddish-purple coloration were identified as presumptive *E. coli*. The representatives of these presumptive *E. coli* colonies were subsequently confirmed using the Vitek^®^ MS mass spectrometry microbial identification system (BioMérieux, Marcy L’Etoile, France; analysis conducted by KVDC testing laboratory, Kasetsart University, Kamphaeng Saen Campus). The averages of colonies or confirmed colonies were calculated as CFU and log CFU per gram of sample.

#### 2.5.4. Analysis of Bacterial Pathogens

The enumeration of *Staphylococcus aureus*, *Bacillus cereus*, and *Clostridium perfringens* was conducted utilizing modified plate count methods described in Chapters 12, 14, and 16 of the US FDA’s BAM, respectively [29,30,31]. A 0.2 mL sample from each dilution was surface-spread in duplicate on Baird Parker agar supplemented with Rabbit Plasma Fibrinogen (BP-RPF), Bacara^®^ agar, and Tryptose Sulfite Cycloserine (TSC) agar with TSC overlay. All media were supplied by BioMérieux, Marcy L’Etoile, France. The BP-RPF plates were incubated for 48 h at 37 °C, Bacara plates for 24 h at 30 °C, and TSC plates for 24 h at 37 °C under anaerobic conditions using GENbag anaer sachets from BioMérieux, Marcy L’Etoile, France. Subsequently, presumptive colonies of *S. aureus*, presumptive *B. cereus*, and *C. perfringens* were enumerated. In the case of *S. aureus*, colonies appeared black and were surrounded by an opaque zone, which may or may not have an outer clear zone on BP-RPF. Presumptive *B. cereus* colonies exhibited pink or orange coloration, encircled by an opaque zone on Bacara. *C. perfringens* exhibited black colonies measuring 2–4 mm, surrounded by opaque zones on TSC.

*Salmonella* spp. were examined through the presence/absence (P/A) test. A 25 g portion of each frozen insect sample was enriched in Buffered Peptone Water (BPW) supplemented with BioMérieux *Salmonella* Supp Tab and incubated at 41.5 °C for 16–24 h. A portion of the enrichment medium was streaked onto SALMA agar (BioMérieux, Marcy L’Etoile, France), and the plate was incubated at 37 °C for 21 to 27 h. Presumptive *Salmonella* colonies exhibit a purple coloration, differentiating them from the blue colonies of other bacteria.

Presumptive colonies of *S. aureus*, presumptive *B. cereus*, *C. perfringens*, and *Salmonella* were confirmed using the Vitek^®^ MS mass spectrometry microbial identification system (BioMérieux, Marcy L’Etoile, France), with analysis conducted by the KVDC testing laboratory at Kasetsart University (Kampaengsaen campus). The confirmed quantities of *S. aureus*, presumptive *B. cereus*, and *C. perfringens* were determined as CFU and log CFU per gram of sample. *Salmonella* spp. was determined as either detected (present) or not detected (absent) in 25 g of the sample.

## 3. Results and Discussion

### 3.1. Analysis of Bacterial Diversity Using 16S rRNA Gene High-Throughput Sequencing

Following the processing of FASTQ files derived from high-throughput sequencing of the 16S rRNA gene with the DADA2 package, a total of 3.2 million raw paired-end reads were produced across the five insect species studied (*n* = 6 per species). The read counts varied between 82,837 and 127,355, yielding an average of 108,282 per sample. Following the processes of filtering, denoising, and chimeric sequence removal, an average of approximately 89,092 amplicons was obtained (Table S1). Rarefaction analysis was conducted to identify ASVs, and the resulting rarefaction curves demonstrate adequate representation of the microbial community in each sample (Figure S1). According to microbial analysis based on the 16S rRNA gene, microbial taxa detected in this study were classified into 30 phyla, 62 classes, 137 orders, 248 families, 587 genera, and 6064 ASVs.

**Figure 2:**
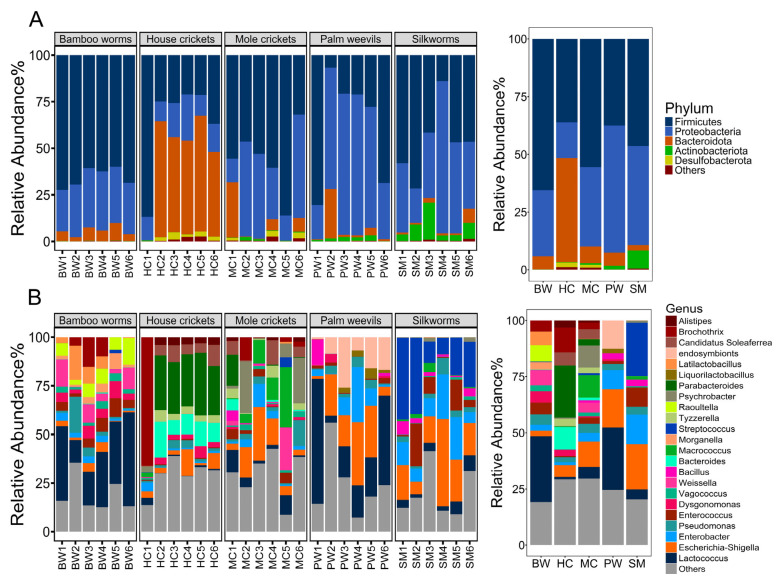
Distribution of the relative abundance of the major bacterial phyla (**A**) and genera (**B**) in different types of edible insects: bamboo worms (BW), house crickets (HC), mole crickets (MC), palm weevils (PW), and silkworms (silk moth larvae) (SM) (*n* = 6 for each type). For each taxon, the relative abundance of ASVs for individual samples and the average for each insect type are shown.

Edible insects contain varied bacterial communities, with their composition influenced by multiple factors. The taxonomic distribution of abundant bacteria across the six samples of each edible insect type at the phylum level is illustrated in Figure 2A. A significant number of dominant ASVs (>2% relative abundance) were identified across the samples. Taxonomic analysis indicated that the majority of sequences in all samples were associated with the phyla Firmicutes (Bacillota), Proteobacteria (Pseudomonadota), Bacteroidota, and Actinobacteriota (Actinomycetota).

Firmicutes was the most abundant phylum in BW, MC, and SM, with proportions of 65.60%, 55.60%, and 46.40%, respectively. This was followed by Proteobacteria at 28.60% in BW, 34.30% in MC, and 42.90% in SM. Bacteroidota accounted for 5.58% in BW and 7.26% in MC, while Actinobacteriota represented 7.79% in SM. Proteobacteria constituted 55.00% of PW microbiota, while Firmicutes accounted for 37.60%. In HC, a well-studied insect, the highest relative abundance was attributed to Bacteroidota (45.20%), followed by Firmicutes (36.10%) and Proteobacteria (15.40%) (Figure 2A and Table 1). The findings align with those reported by Vandeweyer et al., (2017) and Garofalo et al., (2017), indicating that Firmicutes, Bacteroidota, and Proteobacteria are the predominant phyla in HC [32,33].

The percentages of relative abundance of bacterial genera in the individual samples (which were from different sources) and in each insect type are shown in Figure 2B and Table 2. At the genus level, the distribution varied by insect type. *Lactococcus* was the predominant genus in BW and PW (29.37% and 27.73%). It had also been previously found in other insects, such as mealworms and crickets [32,33], although not in such a high proportion as found in our study. The most abundant genus observed in HC was *Parabacteroides* (23.21%). *Bacteroides* (9.80%) was another predominant genus that had been found in most HC samples. MC was dominated by *Escherichia-Shigella* (11.28%), *Macrococcus* (9.96%), and *Psychrobacter* (9.82%). The most abundant genera observed in SM were *Streptococcus* (23.92%), *Escherichia-Shigella* (20.15%), *Enterobacter* (13.06%), and *Enterococcus* (8.53%) (Table 2). Additionally, Chen et al. (2018) had also previously reported that *Enterococcus* and *Enterobacter* were among the most abundant genera that dominated silkworms’ gut microbiome [34]. However, *Streptococcus* and *Escherichia-Shigella* are not typically reported as the most abundant bacteria in the healthy silkworm *Bombyx mori* gut.

Some bacterial genera are uniquely associated with specific insect species. The core microbiomes exhibited distinct differences between HC and MC. The results indicated that *Parabacteroides* and *Bacteroides* were distinct genera in the HC samples. This aligns with prior research indicating that *Parabacteroides* and *Bacteroides* are the predominant genera in the house cricket species (*A. domesticus*) [35]. A separate study identified *Parabacteroides* in certain samples of house crickets [32]. *Lactococcus* was the predominant genus in the bacterial population during the larval stage (BW and PW). The elevated relative abundance of *Lactococcus* in the BW and PW larvae is notably different from the other insect samples analyzed in our study. The findings align with those reported in mealworms and their frass during the complete larval development phase [36]. The notable prevalence of *Streptococcus* appears to be specific to the SM samples analyzed in this study, potentially influenced by factors such as feed, rearing methods, and climatic conditions. Specific species and strains of *Lactococcus* and *Streptococcus* may provide health advantages, since certain members have probiotic characteristics, hence enhancing the functional aspects of food products derived from BW, PW, and SM insects.

Overall, bacterial genera are evidently diverse across different species of edible insects, and they can form complex ecosystems [37]. Significant proportions (19.14–29.66%) of other genera, each with less than 1% abundance, were observed in the examined insect species, indicating a greater diversity of bacterial genera present in these insect species.

### 3.2. Bacterial Community Diversity and Structure

**Figure 3:**
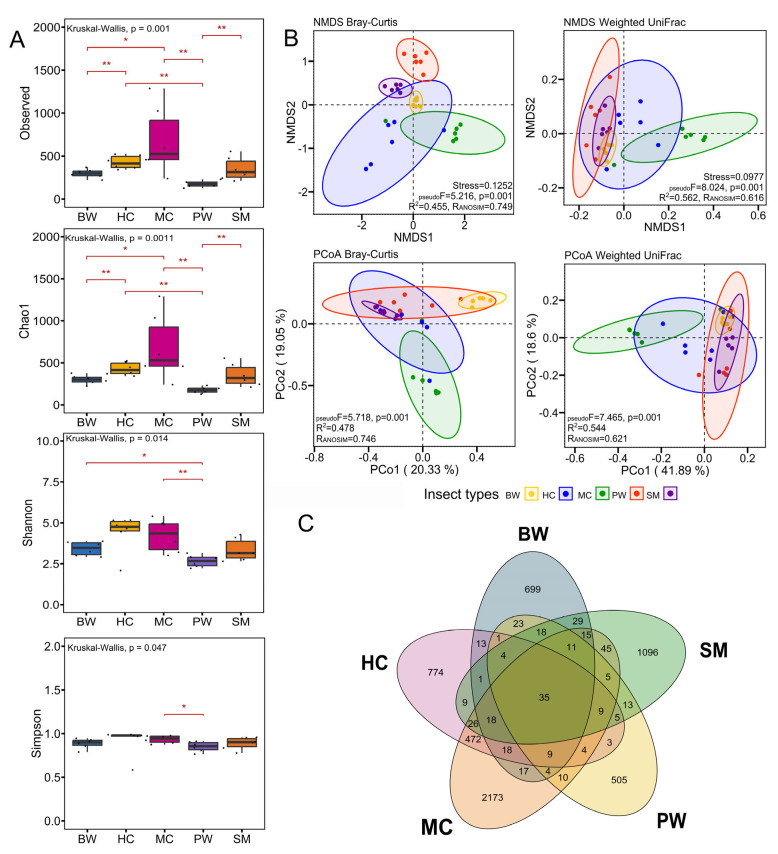
Bacterial diversity (alpha and beta diversity) and core microbiota in the following five types of frozen edible insects tested in this study: bamboo worms (BW), house crickets (HC), mole crickets (MC), palm weevils (PW), and silkworms (silk moth larvae) (SM) (*n* = 6 for each type). (**A**) Alpha diversity index plots based on Observed, Chao1, Shannon, and Simpson. Significant differences were determined using Kruskal-Wallis and Wilcoxon rank sum tests; *p* < 0.05 (*) and *p* < 0.01 (**). (**B**) NMDS and PCoA represent beta diversity with Bray-Curtis and weighted UniFrac dissimilarity. Stress: two-dimensional stress level on the plot; R^2^: retrieved from PERMANOVA test (Adonis); R: retrieved from ANOSIM; values indicating the degree of separation between groups from Analysis of Similarity; (**C**) Venn diagrams showing the ASVs’ distributions.

The α-diversity of the bacterial community varied among different insect types (Figure 3A). The Observed and Chao1 indices indicated that MC exhibited the highest bacterial community richness, succeeded by HC. The lowest bacterial community richness was recorded in PW, with all groups exhibiting significant differences in bacterial community richness (Kruskal-Wallis, *p* = 0.001). The Shannon diversity index showed a comparable trend; however, the differences among the groups were significantly reduced (Kruskal-Wallis, *p* = 0.014). The analysis of the Simpson index indicated significant differences among all insect groups (Kruskal-Wallis, *p* = 0.047); however, significant differences were observed only between the MC and PW groups. The diversity of the HC and MC microbiomes was greater than that of the other insect groups, while the PW microbiome exhibited the lowest diversity among the tested insect groups.

Assessment of beta diversity using both Bray-Curtis and weighted UniFrac matrices indicated that insect type was a strong driver of both microbial community structure and the presence/absence of microbes across the samples when assessed independently. Distance-based PERMANOVA analyses (Bray-Curtis and weighted UniFrac distances) indicated that insect type was a strong driver of microbial community composition among samples. To visualize these relationships, ordination analyses, NMDS, and PCoA were used (Figure 3B and Table S2). The NMDS ordination based on Bray-Curtis and weighted UniFrac distance showed that the microbiome structures were very diverse in specific habitats. Furthermore, the PCoA of Bray-Curtis and weighted UniFrac distance showed that the distributions of microbiomes were significantly separated by insect groups based on the first and second components of the total variation, demonstrating 39.38 and 60.49%, respectively (Figure 3B, see also Supplementary Tables S2–S4). These observations indicate that specific bacterial compositions are associated with particular insect types in this cohort. The analyses of the ASVs that are common to the five types of edible insects are shown in Figure 3C. A total of 35 ASVs were detected as core microbiota and shared between groups. This could be affected by insect species, rearing, and the production process [8].

### 3.3. Relative Abundance of Selected Microorganisms at Family Levels

**Figure 4:**
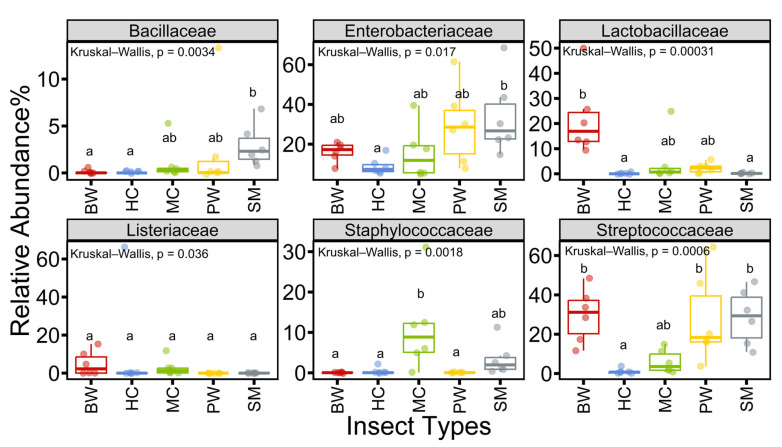
Relative abundance percentages of ASVs at the family level in frozen edible insects (BW: bamboo worms, HC: house crickets, MC: mole crickets, PW: palm weevil larvae, and SM: silkworms (silk moth larvae)). The analysis focused on important microbial indicators belonging to the following six taxonomic families: *Bacillaceae*, *Enterobacteriaceae*, *Lactobacillaceae*, *Listeriaceae*, *Staphylococcaceae*, and *Streptococcaceae*. Different letters indicate statistically significant differences (*p* < 0.05) among the insect groups according to the Kruskal-Wallis test, followed by Dunn’s test with the Bonferroni adjustment method.

Specific microbial groups play crucial and unique roles in food, serving as indicators of food quality, hygiene, and safety. Total bacterial counts and lactic acid bacteria are indicators of the overall quality of food products, whereas *Enterobacteriaceae* and coliforms indicate hygiene levels, and foodborne pathogens directly reflect food safety [38]. Information regarding these essential microbial indicators is vital for the risk assessment of edible insects and their products, many of which are classified as novel foods. The insect microbiota linked to microbial indicators and foodborne pathogens at the family level offers insights into the quality and safety of these products, as demonstrated by various studies [8]. In our study, the following six families of key microbial indicators associated with food hygiene or safety were analyzed: *Bacillaceae*, *Enterobacteriaceae*, *Lactobacillaceae*, *Listeriaceae*, *Staphylococcaceae*, and *Streptococcaceae*. The findings demonstrated significant differences in the relative abundance of each family across the insect groups (Kruskal-Wallis, *p* < 0.05). *Bacillaceae* exhibited a higher prevalence in SM compared to other types of frozen edible insects, while Enterobacteriaceae were present across all types, with greater abundance in PW and SM relative to the other groups. A notably higher proportion of *Lactobacillaceae* is observed in BW, alongside an increased proportion of *Staphylococcaceae* in MC. *Streptococcaceae* were identified in substantial proportions in BW, SM, and PW. As for *Listeriaceae*, its occurrence was comparatively low in all insect groups (Figure 4 and Table S5).

The relatively high percentages of *Lactobacillaceae* abundance in BW suggest its potential as a significant source of beneficial bacteria. The relatively high proportions of *Streptococcaceae* in BW, SM, and PW correspond with the elevated percentages of *Lactococcus* in BW and PW and *Streptococcus* in SM, as illustrated in Figure 2B. This observation indicates the potential of these insects as a source of beneficial bacteria from the Family *Streptococcaceae*. Some members of the *Streptococcaceae* family are beneficial bacteria, whereas others are pathogenic. The results facilitated the identification of microbiota trends within food ecosystems and the normal bacterial flora in insect samples, as well as bacteria that may be beneficial and/or relevant to food hygiene and safety. The quantitative data on these microbial groups are crucial for identifying necessary actions to enhance hygiene practices, assessing quality management systems in the food industry and evaluating risks to consumer health [39].

### 3.4. Functional Prediction and Analysis of 16S rRNA Gene

**Figure 5:**
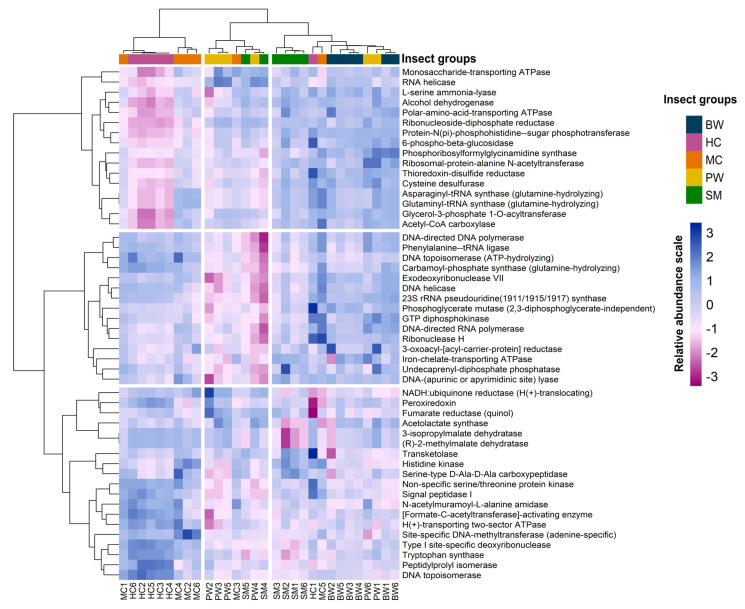
Heatmap visualization of hierarchical clustering of the predicted functions. Samples are categorized according to different insect groups and arranged according to the relative abundance levels. The heatmap color (blue to dark pink) displays the row-scaled relative abundance of each enzyme classification in all samples. Clustering was based on Euclidean, correlation distances (by row and column, respectively), and ward.D2 methods.

Metabolic pathways were predicted by standardizing ASV abundance through PICRUSt2 software and acquiring metabolic enzyme family information related to ASVs via metabolic functions (MetaCyc pathways). The composition and abundance of metabolic functions were predicted using 16S rRNA metagenome data. A clustered heat map was generated to analyze the differences in the abundance of metabolic functions, focusing on the top 50 differential enzyme systems with the highest relative abundance values (Figure 5). A significant number of the predicted enzymes were associated with nutrient–nucleotide metabolism and cellular energy metabolism. The relative abundance of metabolic enzymes is associated with insect types, with distinct patterns of functional distribution observed between juvenile forms (notably BW and SM) and adult forms (HC and MC). This finding suggests that both the composition and function of the microbiome tend to change over the insect life cycle, likely in response to shifts in its ecological niche. Comprehending the mechanisms underlying host–microbiome interactions is essential for investigating host ecology; nevertheless, they remain inadequately defined [40,41]. Research on *Onthophagus taurus* has shown that the host supported a very diversified microbiota, which experienced significant community alterations during its development. The host’s developmental stage, exposure to environmental microbiota, and, to a lesser extent, sex, primarily influence these changes [40].

**Figure 6:**
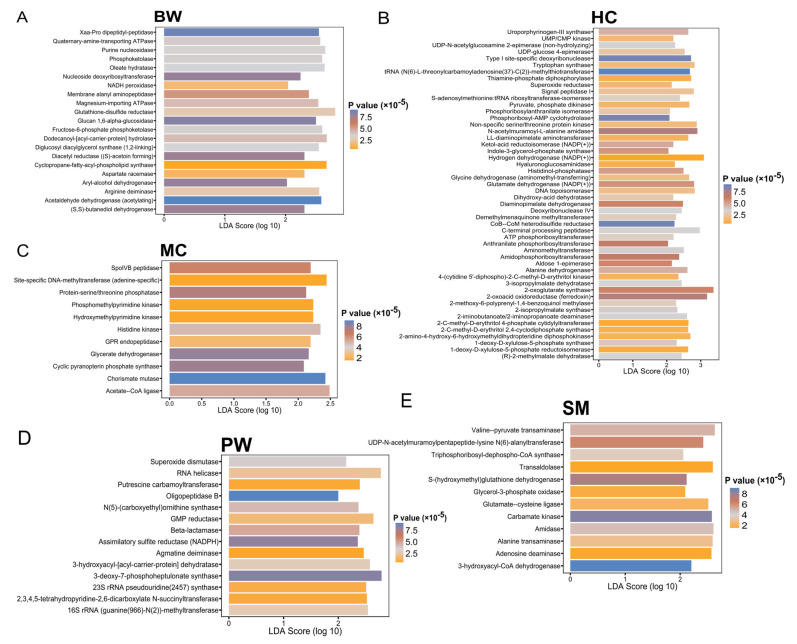
Sets of functional pathways (MetaCyc) predicted by PiCrust2, which are associated with the following insect groups: (**A**) bamboo worms (BW), (**B**) house crickets (HC), (**C**) mole crickets (MC), (**D**) palm weevil larvae (PW), and (**E**) silkworms (silk moth larvae) (SM). Differentiating pathways were identified using LEfSe with LDA effect size ≥ 2 and alpha ≤ 0.01.

To enhance the understanding of the potential impacts of taxonomic structure variations within each insect group on functional pathways, metagenomic diversity was predicted, and differential abundance in predicted metabolic functions was identified (MetaCyc pathways). Further profiles were identified through LDA effect size (LEfSe) analyses, revealing 22 pathways for BW, 44 for HC, 12 for MC, 17 for PW, and 30 for SM that distinguish the predicted functional profiles of the insect groups (LDA effect size ≥ 2 and alpha ≤ 0.01) (Figure 6). LEfSe analysis is essential for identifying statistically robust and biologically meaningful biomarkers in edible insect bacterial metagenomics by combining a strong significance threshold (*p* < 0.01) with a high effect size (LDA > 2.0) [22]. This approach facilitates the distinction of microbial enzyme functions and biomarkers that could affect food safety, nutritional value, or processing quality. The metabolic functions observed are largely attributed to the gut microbiota, which encode various metabolism-related enzymes influenced by the host diet and environment [42]. These microbes play key roles in nutrient provision and the degradation of complex molecules through symbiotic relationships [42,43,44]. In this study, we found that BW were enriched in peptidases and ATPases, indicating enhanced protein digestion and ion transport, whereas HC exhibited greater diversity in transferases and dehydrogenases, consistent with their plant-based diet. MC had higher levels of DNA-modifying enzymes, reflecting adaptability to a soil-associated habitat. PW exhibited antioxidant enzymes, including superoxide dismutase, which facilitate oxidative stress tolerance, whereas SM displayed elevated transaminase and dehydrogenase activities, indicating active amino acid and carbohydrate metabolism. Overall, these findings show that gut microbial communities in edible insects are functionally specialized according to host diet and ecological niche, potentially affecting nutritional quality and safety [45,46,47].

### 3.5. Microbiological Analysis

Since metagenetic data (relative abundance of ASVs at the family level) indicated the presence of certain key microbial groups that might be related to quality and safety, the samples of frozen edible insects were then subjected to the culture-based analysis of specific microbial indicators. Considering the metagenetic data that pointed to the presence of certain key microbial indicators, results from a previous study [37], together with existing microbiological criteria of food products of similar types [48,49], microbiological analysis in this study was designed to cover microbial indicators for general quality (TVC, LAB, YM, and SFB), indicators for hygiene condition (*Enterobacteriaceae* and *E. coli*), and some key bacterial foodborne pathogens (presumptive *B. cereus*, *C. perfringens*, *S. aureus*, and *salmonella* species).

The counts of TVC, LAB, YM, SFB, *Enterobacteriaceae*, and *E. coli* exhibited significant variability across samples. As for foodborne bacterial pathogens, presumptive *B. cereus* was found in different levels in some samples, whereas *C. perfringens* and *S. aureus* were below the limit of quantitation in all samples, and *Salmonella* spp. was not present in any of the samples analyzed (Table 3 and Table 4).

Significant differences at a 95% confidence level were observed in TVC, SFB, and *B. cereus* across different frozen insect types (Table 4). The average total TVC ranged from 3.41 ± 0.76 to 5.19 ± 0.79 log CFU/g, with the values for SM significantly exceeding those of other edible insect types (Table 4). The analysis of individual samples revealed that several frozen insect samples exhibited TVC levels exceeding 5.0 log CFU/g, surpassing the threshold specified in criteria for frozen edible insects, such as house crickets and mealworms [48,49]. The diverse TVCs identified for certain frozen insects suggest potential influences from factors beyond insect type, including rearing and processing methods, hygiene, and storage conditions [17,50].

The LAB counts varied from 4.02 ± 1.43 to 5.87 ± 0.46 log CFU/g (Table 4), comparable to levels observed in insect powder samples (house crickets and mealworms) [51] and frozen edible insects (silkworms, bamboo caterpillars, and field crickets) [52]. The findings indicate that edible insects may provide a significant source of beneficial bacteria, supporting the results of metagenetic analysis that identified high levels of relative abundance of genus *Lactococcus* and families *Lactobacillaceae* and *Streptococcaceae* in certain edible insect species (Figure 4). Lactic acid bacteria are naturally found in insects and can play an important role in fermentation and shelf-life extension. They can contribute to the bioactive properties of fermented edible insect products [53]. This group of bacteria also serves as a significant quality indicator; particularly, elevated levels of LAB could indicate the spoilage status of non-fermented food products. Some lactic acid bacteria, such as *Pediococcus pentosaceus*, *Enterococcus faecium*, and *Lactobacillus* spp., had probiotic properties and can contribute to stabilizing gut microflora, combating pathogens, and improving nutrient use in some insects [54,55].

Yeast and mold counts serve as indicators for the general quality of food. It serves as an indicator of spoilage status, as elevated yeast and mold counts may correlate with off-flavors or alcohol flavors in food products. The yeast and mold counts observed in our study ranged from 2.94 ± 0.96 to 4.41 ± 0.97 log CFU/g (Table 4). Nyangena et al., (2020) reported that the concentrations of yeasts and molds in raw edible insects ranged from 7.7 to 9.1 log CFU/g [17]. A separate study indicates that yeast and mold counts in frozen edible insects range from 5.59 to 6.27 log CFU/g [52]. The lower levels observed in our study were likely due to processing; however, the majority of samples still exhibited yeast and mold counts exceeding the limits established in European criteria for frozen edible insects (≤100 CFU/g or ≤2.00 log CFU/g) [48,49].

Regarding SFB, the levels varied from 2.23 ± 0.28 to 3.84 ± 0.72 log CFU/g (Table 4), with mole crickets exhibiting the highest number of SFB among the tested edible insect types. Aerobic spore-forming bacteria are frequently identified in various edible insects [56]. The problems with SFB in food preservation arise from the widespread presence of microbial spores and their remarkable resistance to various environmental stresses. Because these spores can endure standard food processing methods, they present a major concern for food quality and safety within the food industry [57]. Therefore, thermal and non-thermal processing methods should be well-designed to reduce the number of SFB.

*Enterobacteriaceae* and *E. coli*, two key hygiene indicators generally established in international food regulations, were detected at concentrations ranging from <1.00 to 5.05 log CFU/g and <1.00 to 2.70 log CFU/g, respectively (Table 4). The European criteria for frozen edible insects (house crickets and mealworms) specify that the levels of *Enterobacteriaceae* and *E. coli* should not exceed 100 and 50 CFU/g, respectively [48,49]. The findings of this study indicate a potential necessity for enhanced hygiene practices in the manufacturing of these frozen edible insects.

Among foodborne bacterial pathogens, *B. cereus* is one of the most frequently contaminating pathogens in many types of foods. *B. cereus* can cause gastrointestinal illnesses from the two forms of toxin it produces—enterotoxins and emetic toxin [58]. Recently, some food criteria require the examination of *B. cereus* group or presumptive *B. cereus* instead of *B. cereus* (or *B. cereus* sensu stricto). This is because some members of closely related species in the *B. cereus* group, particularly *B. thuringiensis* and *B. paranthracis*, have been associated with gastrointestinal illnesses and have been recognized as foodborne pathogens [59,60]. Some closely related species can be isolated in the laboratory in the step of selective isolation and are indistinguishable from *B. cereus*; therefore, isolates obtained in this step are referred to as presumptive *B. cereus* [30]. In many cases, it is counted as sufficiently relevant information, since confirmatory tests based on biochemical reactions are inadequate to distinguish between *B. cereus* and other closely related species [61].

In our study, presumptive *B. cereus* varied from <1.70 to 3.93 log CFU/g, with the maximum count detected in silkworms (Table 4). Presumptive *B. cereus* counts in some samples exceeded the limit (≤2.00 log CFU/g) set in Regulations (EU) 2022/188 and 2022/169 [48,49]. However, our findings fall within the range (2.0 to 6.6) of presumptive *B. cereus* previously found across various edible insect species, including crickets, mole crickets, silkworms, and mealworms [56]. *B. cereus* is widely distributed in the environment and commonly contaminates many types of foods. Its endospores can survive harsh conditions, such as extreme temperatures and pH, both in the natural environment and food manufacturing [58,61]. Moreover, strains of *B. cereus* can produce biofilm that can cross-contaminate foods, leading to a short shelf life and increased risks of foodborne illnesses [62].

Other foodborne pathogens associated with food of animal origin are *C. perfringens*, *S. aureus*, and *Salmonella.* All samples were under the quantitation limit (<50 CFU/g or <1.7 log CFU/g) for *C. perfringens* and *S. aureus* (Table 4), and all were negative for *Salmonella*, indicating low risks of staphylococcal food poisoning and *Clostridium perfringens* and *Salmonella* gastroenteritis associated with frozen edible insects. Nevertheless, it remains essential to monitor these pathogens, as prior studies have recorded the presence of *Clostridium* in diverse insect species using uncultured methods [16,63,64], and *C. perfringens* spores could survive in frozen food samples, although vegetative cells were not tolerant to freezing temperatures [65]. Furthermore, *S. aureus* was previously detected in ready-to-eat mopani worms (*Gonimbrasia belina*) [66], and *S. aureus* and *Salmonella* species have been found in raw edible insects, including black soldier fly, cricket, grasshopper, and African cotton leafworm [17].

**Figure 7:**
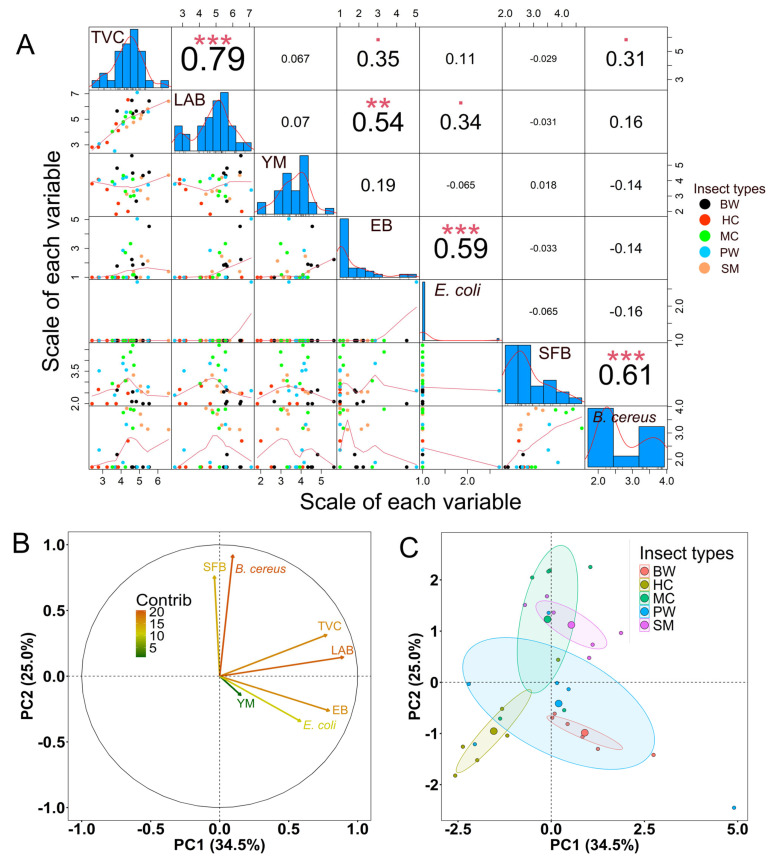
Correlation analysis between microbial groups in five types of frozen edible insects. (**A**) The scatterplot matrix comparing pairs of variables of interest with red trend lines is shown in the lower part of the diagonal boxes of microbiological tests. The upper triangular panel (above the diagonal boxes of the histogram) indicates the number representing the correlation coefficient between each variable. Pearson correlations for comparisons between two variables are shown above the diagonal boxes. The correlation coefficient values in larger font sizes indicate more significant correlations (▪ *p* < 0.05, ** *p* < 0.001, and *** *p* < 0.0001). TVC: total viable counts, LAB: lactic acid bacteria, YM: yeasts and molds, EB: *Enterobacteriaceae*, SFB: spore-forming bacteria. (**B**) Principal component 2D biplot showing the distribution of variable vectors inside a unit circle. (**C**) Principal component analysis (PCA) for various microbial detections of individual samples in different insect categories. Abbreviations: BW: bamboo worms, HC: house crickets, MC: mole crickets, PW: palm weevil larvae, and SM: silkworms (silk moth larvae).

The pairwise relationships among levels of microbial groups or types were analyzed and are shown in Figure 7A. A significant correlation was observed between TVC and LAB (*p* < 0.0001), TVC and *Enterobacteriaceae* (*p* < 0.05), as well as TVC and presumptive *B. cereus* (*p* < 0.05). A strong positive correlation was observed between LAB *and E. coli* (*p* < 0.05), as well as between LAB and *Enterobacteriaceae* (*p* < 0.001). Furthermore, a strong correlation was identified between *Enterobacteriaceae* and *E. coli* (*p* < 0.0001), as well as between SFB and presumptive *B. cereus* (*p* < 0.0001).

Principal component analysis (PCA) was employed to establish correlations among the groups or types of microbes. A two-dimensional scores plot was generated from the variation factors utilizing the first and second principal components (PC1 and PC2), accounting for 34.5% and 25.0% of the variance in the dataset, respectively (Figure 7B). TVC, LAB, *Enterobacteriaceae*, and *E. coli* exhibited strong positive correlations with PC1, while SFB and presumptive *B. cereus* demonstrated significant positive correlations with PC2. The PCA indicated that the frozen edible insects exhibit comparable microbial loads within groups (Figure 7C). The edible insects in the larval stage, including bamboo worms and palm weevil larvae, exhibit a clear overlap (Figure 7C), while house crickets and mole crickets were distinctly grouped based on their microbial loads.

These findings indicate variability in microbial loads and microbiological qualities, including hygiene and safety potentials, among frozen insect samples. Besides insect types, such variability can depend on insect feed, rearing environment, and processing methods.

In Thailand, the cultivation of edible insects utilizes locally sourced food resources. Bamboo worms are cultivated within bamboo and sustain themselves on bamboo pulp. House crickets are fed with plant matter or commercial animal feedstuffs. Mole crickets are raised with plant matter and/or fresh grass. Nonetheless, other food sources or organic materials may be used in small-scale farming. Palm weevil larvae consume the pith or soft tissue of coconut trees (or animal feedstuffs in contemporary farming), while silkworms feed on mulberry leaves. Although these are common practices for feeding, it should be noted that the feed provided may be different in different geographical areas, farms, and seasons. Different microbes in these feed varieties can influence microbial communities in different types of insects, rearing areas, and individual insect farms.

Once harvested, edible insects are processed into insect food products either in household- or industrial-scale production. Typically, farm-raised insects are dispatched for processing. Fasting often occurs one to three days prior to harvesting. The insects undergo raw material screening and washing. They are then immersed in cold water (ca. 4 °C or lower), blanched at 95–100 °C for 10 min, washed with cold water again, and then drained. The insects are frozen using either individual quick freezing (IQF) or gradual freezing at around −18 °C; the latter method is employed in household-scale production. The products are stored at −18 to −20 °C. The methods of processing can also vary, contributing to the differences in microflora in edible insects. Processing hygiene conditions can also play a significant role in the determination of microbiological quality.

Microbial contamination can be ascribed to inadequate hygiene practices and poor sanitation during harvesting and processing, fecal contamination, improper storage, and insufficient cooking [67]. The post-harvesting process of edible insects can substantially influence their safety and quality. Insects should undergo a starvation phase to decrease microbial loads from intestinal fecal matter, which is crucial for mitigating food safety hazards and improving the quality of final products [68,69]. The results presented in Table 3 indicate that it is possible to apply the stringent control of rearing, processing, and good hygiene practice, so that edible insect products will have microbiological qualities that comply with available standards. Good practice in edible insect production throughout the supply chain and scientific-based microbiological criteria will provide a clear guideline and framework for food producers.

In addition, the increase in insect farming and the expansion of international trade of edible insect products depend heavily on regulatory frameworks. Establishing clear international policies and legislation, standardization, and certification processes is particularly critical for international commerce. Addressing microbiological quality and safety of edible insect products, diets and rearing methods, genetic modifications, and potential health risks, such as digestibility, toxicity, and allergenicity, are key considerations in formulating regulatory frameworks [70]. Therefore, our findings, which demonstrate the microbial safety of edible insects, provide valuable evidence to inform regulatory policies and support the export of these products to global markets.

However, limitations of this study were acknowledged. The presence of uncontrolled environmental variables, such as contamination, temperature, humidity, transportation, handling procedures, storage, and safety could influence the microbial community composition study. Additionally, the edible insect studies may be affected by strain, age, sex, diet, housing conditions, breeding, and several other factors [71]. Future research utilizing controlled environmental conditions could strengthen the reliability of outcomes. Recognizing these limitations can guide future research efforts, enhancing the overall reliability and applicability of microbiological and metagenomic investigations.

## 4. Conclusions

This study applied a culture-independent method to investigate and compare bacterial populations in five types of commercially available frozen edible insects from different sources. Although some bacterial taxa (Firmicutes (Bacillota) and Proteobacteria (Pseudomonadota)) were common among all insect types, there were distinct predominant taxa in each type, and overall, the microbiomes were diverse in different types of edible insects. The results also indicated their potential roles as sources of beneficial bacteria, while also stressing the necessity to monitor the quality and safety of these frozen insect products. Culture-dependent microbiological analysis indicated varied levels of microbial groups, encompassing general quality indicators, hygiene indicators, and foodborne pathogens across various samples of each insect type. The analysis revealed significant correlations among various microbial groups, with particularly strong associations observed between TVC and LAB, SFB and presumptive *B. cereus*, as well as among *Enterobacteriaceae* and *E. coli*. Principal component analysis indicated that most frozen edible insects exhibit slightly different microbial profiles within their respective groups. Larval insects (bamboo worms and palm weevil larvae) showed complete overlap in their microbial communities, whereas house crickets and mole crickets displayed distinct microbial profiles. In certain samples, the microbial loads of general and specific microbial groups, especially hygiene indicators and a foodborne pathogen, did not meet the acceptable levels outlined in microbiological criteria (EU regulations), which raises concerns regarding their quality and safety. To enhance the microbiological quality and safety of frozen edible insects, guidelines for good practice in edible insect farming should be given. Guidelines for the manufacturing of edible insects and hygiene practice, which will promote the stability of product microbiological quality and ensure safety, should be established. Advanced heat treatment protocols should be implemented to effectively reduce microbial loads, particularly targeting *B. cereus* group and other potential pathogens. Future international food safety and commerce agreements should include specific provisions for edible insect trade. Investing in innovative packaging solutions that maintain product microbiological quality during storage and transport is crucial.

This research lays the groundwork for the continued exploration of edible insects as a sustainable and nutritious food source, while emphasizing the importance of rigorous safety standards in this emerging industry. A point to be considered for the future study is the long-term health impacts of regular edible insect consumption, as well as exploring the use of beneficial bacteria or potential probiotics found in edible insects for the enhancement of functions and health promotion. A potential improvement for future research is the incorporation of controlled environmental conditions, which could further clarify their influence on microbial communities. Establishing comprehensive microbial monitoring and testing programs throughout the supply chain is essential to ensure consistent quality and safety. To facilitate seamless international trade, globally recognized safety and quality benchmarks for edible insects should be developed. Existing criteria can be adopted, such as EU regulations, but they will be subject to periodic revision as more insect types are being considered as novel food, new processing methods are being employed, and new products are being developed.

## Figures and Tables

**Table 1:** Statistical analysis of relative abundance of several bacterial phyla in edible insect group using Kruskal-Wallis and Dunn’s test for post hoc tests.

Insect Group	Relative Abundance (%)					
	Firmicutes	Proteobacteria	Bacteroidota	Actinobacteriota	Desulfobacterota	Others
BW	65.60 ± 5.20	28.60 ± 3.59 ^ab^	5.58 ± 2.56 ^ab^	0.09 ± 0.05 ^a^	0.04 ± 0.02 ^ab^	0.10 ± 0.10 ^a^
HC	36.10 ± 25.50	15.40 ± 5.45 ^a^	45.2 ± 23.1 ^b^	0.11 ± 0.17 ^a^	2.01 ± 1.30 ^a^	0.18 ± 1.14 ^b^
MC	55.60 ± 19.90	34.30 ± 18.90 ^b^	7.26 ± 11.4 ^a^	0.65 ± 0.66 ^ab^	1.29 ± 1.48 ^a^	0.90 ± 1.02 ^b^
PW	37.60 ± 29.70	55.00 ± 24.50 ^b^	5.71 ± 10.3 ^a^	1.58 ± 1.03 ^bc^	0.03 ± 0.04b ^c^	0.09 ± 0.11 ^a^
SM	46.40 ± 19.20	42.90 ± 21.40 ^b^	2.35 ± 2.62 ^a^	7.79 ± 6.51 ^c^	0.00 ± 0.00 ^c^	0.56 ± 0.50 ^b^

Notes: Abbreviations: BW: bamboo worms, HC: house crickets, MC: mole crickets, PW: palm weevils, and SM: silkworms (silk moth larvae). Values given as means ± SD. Different letters across columns indicate statistically significant differences (*p* < 0.05) in percentages of relative abundance among the insect groups (Kruskal-Wallis and Dunn’s tests).

**Table 2:** Relative abundance of bacterial genera in five frozen edible insect groups.

BW		HC		MC		PW		SM	
Genus	% Relative Abundance	Genus	% Relative Abundance	Genus	% Relative Abundance	Genus	% Relative Abundance	Genus	% Relative Abundance
*Lactococcus*	29.37 ± 13.43	Others	29.31 ± 8.43	Others	29.66 ± 12.34	*Lactococcus*	27.73 ± 22.68	*Streptococcus*	23.92 ± 14.11
Others	19.14 ± 9.18	*Parabacter-oides*	23.21 ± 11.62	*Escherichia-Shigella*	11.28 ± 9.65	Others	24.60 ± 17.01	Others	20.33 ± 13.12
*Raoultella*	7.28 ± 3.66	*Bacteroides*	9.80 ± 6.03	*Macrococcus*	9.96 ± 11.55	*Escherichia-Shigella*	17.01 ± 12.22	*Escherichia-Shigella*	20.15 ± 13.09
*Weissella*	6.72 ± 5.60	*Candidatus Soleaferrea*	5.89 ± 3.65	*Psychrob-acter*	9.82 ± 12.12	*Endosymb-ionts*	12.54 ± 8.90	*Enterobacter*	13.06 ± 7.57
*Latilactob-acillus*	6.05 ± 6.56	*Alistipes*	3.14 ± 1.57	*Lactococcus*	5.17 ± 4.60	*Enterobacter*	8.55 ± 10.47	*Enterococcus*	8.53 ± 8.77
*Enterococcus*	5.24 ± 2.66	*Tyzzerella*	3.28 ± 1.90	*Candidatus* *Soleaferrea*	4.36 ± 5.02	*Pseudomonas*	2.78 ± 2.05	*Lactococcus*	4.5 ± 2.46
*Dysgonom-onas*	5.02 ± 2.40	*Escherichia-Shigella*	5.23 ± 4.83	*Weissella*	4.32 ± 8.64	*Bacillus*	2.47 ± 5.26	*Pseudom-onas*	3.47 ± 2.55
*Brochothrix*	4.94 ± 6.38	*Dysgonom-onas*	2.77 ± 1.95	*Pseudomonas*	3.96 ± 4.71	*Liquorilactobacillus*	2.11 ± 1.97	*Bacillus*	2.9 ± 2.23
*Pseudomonas*	4.59 ± 6.93	*Pseudomonas*	2.15 ± 1.55	*Enterobacter*	3.90 ± 4.12	*Enterococcus*	1.21 ± 1.03	*Macrococcus*	1.44 ± 1.75
*Morganella*	3.36 ± 2.73	*Enterobacter*	1.40 ± 1.58	*Brochothrix*	2.86 ± 4.57	*Dysgonom-onas*	0.91 ± 1.79	*Psychrob-acter*	0.88 ± 1.02

Notes: Abbreviations: BW: bamboo worms, HC: house crickets, MC: mole crickets, PW: palm weevils, and SM: silkworms (silk moth larvae). Values given as means ±SD.

**Table 3:** Results of microbiological analysis of frozen edible insects by sample.

Type of Frozen Edible Insect	Source of Sample	General Quality Indicator				Hygiene Indicator		Bacterial Pathogen			
		Total Viable Count (CFU/g)	Lactic Acid Bacteria (CFU/g)	Yeasts and Molds (CFU/g)	Spore-Forming Bacteria (CFU/g)	*Enterob-acteriaceae* (CFU/g)	*E. coli* (CFU/g)	Presumptive *B. cereus* (CFU/g)	*C. perfringens* (CFU/g)	*S. aureus* (CFU/g)	*Salmonella* (in 25 g)
Bamboo worms	Store A	6.60 × 10^4^	4.41 × 10^5^	4.67 × 10^2^	1.25 × 10^2^	<1.00 × 10^1^	<1.00 × 10^1^	<5.00 × 10^1^	<5.00 × 10^1^	<5.00 × 10^1^	ND
	Store B	3.80 × 10^4^	3.03 × 10^6^	4.20 × 10^5^	1.25 × 10^2^	1.70 × 10^2^	<1.00 × 10^1^	<5.00 × 10^1^	<5.00 × 10^1^	<5.00 × 10^1^	ND
	Store C	4.26 × 10^4^	2.86 × 10^5^	3.13 × 10^4^	3.50 × 10^2^	7.00 × 10^1^	<1.00 × 10^1^	5.00 × 10^1^	<5.00 × 10^1^	<5.00 × 10^1^	ND
	Store D	1.53 × 10^5^	3.78 × 10^5^	2.16 × 10^4^	1.00 × 10^2^	8.00 × 10^1^	<1.00 × 10^1^	<5.00 × 10^1^	<5.00 × 10^1^	<5.00 × 10^1^	ND
	Store E	3.30 × 10^5^	2.73 × 10^6^	3.56 × 10^4^	1.00 × 10^2^	3.35 × 10^4^	<1.00 × 10^1^	<5.00 × 10^1^	<5.00 × 10^1^	<5.00 × 10^1^	ND
	Store G	6.00 × 10^3^	4.46 × 10^5^	6.50 × 10^4^	4.25 × 10^2^	6.00 × 10^1^	<1.00 × 10^1^	1.50 × 10^2^	<5.00 × 10^1^	<5.00 × 10^1^	ND
House cricket	Store A	3.00 × 10^4^	3.40 × 10^6^	1.00 × 10^2^	3.00 × 10^2^	<1.00 × 10^1^	<1.00 × 10^1^	5.00 × 10^2^	<5.00 × 10^1^	<5.00 × 10^1^	ND
	Store B	6.96 × 10^3^	1.30× 10^4^	3.33 × 10^2^	6.50 × 10^2^	<1.00 × 10^1^	<1.00 × 10^1^	<5.00 × 10^1^	<5.00 × 10^1^	<5.00 × 10^1^	ND
	Store C	5.30 × 10^3^	4.43 × 10^4^	6.70 × 10^1^	1.00 × 10^2^	<1.00 × 10^1^	<1.00 × 10^1^	<5.00 × 10^1^	<5.00 × 10^1^	<5.00 × 10^1^	ND
	Store D	5.80 × 10^2^	6.67 × 10^2^	1.20 × 10^4^	4.50 × 10^2^	<1.00 × 10^1^	<1.00 × 10^1^	<5.00 × 10^1^	<5.00 × 10^1^	<5.00 × 10^1^	ND
	Store E	1.70 × 10^3^	1.53 × 10^3^	2.66 × 10^3^	1.00 × 10^2^	<1.00 × 10^1^	<1.00 × 10^1^	<5.00 × 10^1^	<5.00 × 10^1^	<5.00 × 10^1^	ND
	Store F	2.60 × 10^2^	6.67 × 10^2^	6.33 × 10^3^	1.00 × 10^2^	<1.00 × 10^1^	<1.00 × 10^1^	<5.00 × 10^1^	<5.00 × 10^1^	<5.00 × 10^1^	ND
Mole crickets	Store B	1.31 × 10^4^	6.35 × 10^4^	2.16 × 10^4^	1.40 × 10^4^	<1.00 × 10^1^	<1.00 × 10^1^	5.93 × 10^3^	<5.00 × 10^1^	<5.00 × 10^1^	ND
	Store C	4.27 × 10^4^	1.51 × 10^5^	1.23 × 10^4^	5.05 × 10^4^	1.50 × 10^1^	<1.00 × 10^1^	1.50 × 10^3^	<5.00 × 10^1^	<5.00 × 10^1^	ND
	Store D	3.30 × 10^3^	1.10 × 10^4^	8.66 × 10^3^	8.50 × 10^2^	<1.00 × 10^1^	<1.00 × 10^1^	<5.00 × 10^1^	<5.00 × 10^1^	<5.00 × 10^1^	ND
	Store E	3.23 × 10^4^	3.59 × 10^5^	9.67 × 10^2^	2.45 × 10^4^	2.15 × 10^3^	<1.00 × 10^1^	7.95 × 10^3^	<5.00 × 10^1^	<5.00 × 10^1^	ND
	Store F	1.70 × 10^4^	1.60 × 10^5^	2.00 × 10^3^	1.15 × 10^3^	5.10 × 10^2^	<1.00 × 10^1^	<5.00 × 10^1^	<5.00 × 10^1^	<5.00 × 10^1^	ND
	Store G	4.91 × 10^4^	1.36 × 10^5^	1.06 × 10^3^	6.00 × 10^3^	1.50 × 10^1^	<1.00 × 10^1^	7.50 × 10^3^	<5.00 × 10^1^	<5.00 × 10^1^	ND
Palm weevil larvae	Store A	7.43 × 10^4^	1.26 × 10^7^	2.46 × 10^3^	4.00 × 10^2^	1.11 × 10^5^	5.05 × 10^2^	<5.00 × 10^1^	<5.00 × 10^1^	<5.00 × 10^1^	ND
	Store D	4.42 × 10^3^	3.33 × 10^2^	6.00 × 10^3^	2.50 × 10^2^	<1.00 × 10^1^	<1.00 × 10^1^	2.50 × 10^1^	<5.00 × 10^1^	<5.00 × 10^1^	ND
	Store E	1.91 × 10^4^	3.63 × 10^5^	1.70 × 10^4^	3.60 × 10^3^	<1.00 × 10^1^	<1.00 × 10^1^	2.06 × 10^3^	<5.00 × 10^1^	<5.00 × 10^1^	ND
	Store F	1.25 × 10^4^	4.34 × 10^4^	1.40 × 10^4^	7.25 × 10^3^	7.25 × 10^2^	<1.00 × 10^1^	7.50 × 10^1^	<5.00 × 10^1^	<5.00 × 10^1^	ND
	Store H	6.33 × 10^2^	1.20 × 10^3^	1.10 × 10^4^	3.30 × 10^3^	<1.00 × 10^1^	<1.00 × 10^1^	2.25 × 10^2^	<5.00 × 10^1^	<5.00 × 10^1^	ND
	Store I	2.93 × 10^5^	3.87 × 10^5^	5.33 × 10^3^	6.00 × 10^2^	<1.00 × 10^1^	<1.00 × 10^1^	2.50 × 10^1^	<5.00 × 10^1^	<5.00 × 10^1^	ND
Silkworms	Store A	1.98 × 10^4^	2.48 × 10^4^	1.33 × 10^3^	1.20 × 10^3^	<1.00 × 10^1^	<1.00 × 10^1^	8.56 × 10^3^	<5.00 × 10^1^	<5.00 × 10^1^	ND
	Store B	2.79 × 10^5^	6.46 × 10^5^	6.67 × 10^2^	3.50 × 10^2^	1.35 × 10^2^	<1.00 × 10^1^	1.36 × 10^3^	<5.00 × 10^1^	<5.00 × 10^1^	ND
	Store C	4.70 × 10^4^	5.76 × 10^4^	2.00 × 10^3^	2.10 × 10^3^	2.50 × 10^1^	<1.00 × 10^1^	7.25 × 10^3^	<5.00 × 10^1^	<5.00 × 10^1^	ND
	Store D	3.77 × 10^6^	2.59 × 10^7^	2.26 × 10^4^	3.00 × 10^2^	2.50 × 10^1^	<1.00 × 10^1^	1.33 × 10^3^	<5.00 × 10^1^	<5.00 × 10^1^	ND
	Store E	1.26 × 10^5^	2.15 × 10^5^	1.60 × 10^4^	3.50 × 10^2^	2.90 × 10^2^	<1.00 × 10^1^	1.97 × 10^3^	<5.00 × 10^1^	<5.00 × 10^1^	ND
	Store G	1.11 × 10^5^	1.15 × 10^5^	2.30 × 10^3^	6.50 × 10^2^	<1.00 × 10^1^	<1.00 × 10^1^	5.18 × 10^3^	<5.00 × 10^1^	<5.00 × 10^1^	ND

Note: ND: not detected.

**Table 4:** Results of microbiological analyses of frozen edible insects.

Microbiological Analysis	Results of Quantitative Analysis (Mean of log CFU/g (Range)) * or Detection					*p*-Value **	Microbiological Limit (log CFU/g) ***
	BW	HC	MC	PW	SM		
General quality indicator							
Total viable count	4.75 ± 0.59 ^ab^	3.41 ± 0.76 ^b^	4.28 ± 0.44 ^ab^	4.20 ± 0.93 ^ab^	5.19 ± 0.79 ^a^	0.0103	≤5.00
	(3.78–5.52)	(2.41–4.48)	(3.52–4.69)	(2.80–5.47)	(4.30–6.58)		
Lactic acid bacteria	5.87 ± 0.46	4.02 ± 1.43	4.99 ± 0.52	4.75 ± 1.71	5.46 ± 1.07	0.0789	NA
	(5.46–6.48)	(2.82–6.54)	(4.04–5.56)	(2.52–7.10)	(4.40–7.41)		
Yeasts and molds	4.41 ± 0.97	2.94 ± 0.96	3.61 ± 0.58	3.89 ± 0.31	3.53 ± 0.61	0.0559	≤2.00
	(2.67–5.62)	(1.83–4.08)	(2.99–4.33)	(3.39–4.23)	(2.82–4.36)		
Spore forming bacteria	2.23 ± 0.28 ^b^	2.32 ± 0.37 ^b^	3.84 ± 0.72 ^a^	3.12 ± 0.6 ^ab^	2.80 ± 0.34 ^ab^	0.0016	NA
	(2.00–2.63)	(2.00–2.81)	(2.93–4.70)	(2.40–3.86)	(2.48–3.32)		
**Hygiene indicator**							
*Enterobacteriaceae*	<1.00–4.53	<1.00	<1.00–3.33	<1.00–5.05	<1.00–2.46	0.1000	≤2.00 (or <2.00)
*Escherichia coli*	<1.00	<1.00	<1.00	<1.00–2.70	<1.00	0.4060	≤1.70
**Bacterial pathogen**							
Presumptive *Bacillus cereus*	<1.70–2.18 ^c^	<1.70–2.70 ^bc^	<1.70–3.90 ^ab^	<1.70–3.32 ^bc^	3.12–3.93 ^a^	0.0063	≤2.00
*Clostridium perfringens*	<1.70	<1.70	<1.70	<1.70	<1.70	NA	NA
*Staphylococcus aureus*	<1.70	<1.70	<1.70	<1.70	<1.70	NA	NA
*Salmonella* (in 25 g)	ND	ND	ND	ND	ND	NA	ND

Note: Abbreviations: BW: bamboo worms, HC: house crickets, MC: mole crickets, PW: palm weevil larvae, and SM: silkworms (silk moth larvae). * Quantitative values are presented as means ± SD (*n* = 6). ND: not detected by qualitative test. Statistical analyses utilized their values equivalent to the limit of quantitation, which was determined to be 1.0 log CFU/g for *Enterobacteriaceae* and *E. coli* and 1.70 log CFU/g for presumptive *B. cereus*, *C. perfringens*, and *S. aureus*. ** Values within a row with different superscripts are statistically significantly different (Kruskal-Wallis, df = 4, *p* ≤ 0.05). Dunn’s test with Bonferroni adjustment. Data values that fell below the quantitation limits were included in the mean calculation. NA: not applicable. *** According to Commission Implementing Regulations 2022/188 (house crickets, *Acheta domesticus*) and 2022/169 (yellow mealworm; *Tenebrio molitor* larva).

## Data Availability

The original contributions presented in the study are included in the article/Supplementary Materials, further inquiries can be directed to the corresponding authors.

## Supplementary Materials

The following supporting information can be downloaded at https://www.mdpi.com/article/10.3390/foods14132347/s1. Figure S1: Rarefaction curves of observed microbial ASVs detected in five frozen edible insect groups; Table S1: Data of raw sequence and sequences obtained after DADA2 was used for denoising, filtering, merging, and chimera removal; Table S2: Statistical analysis of bacterial community structure among edible insect groups; Table S3: Statistical analysis of bacterial community structure between frozen edible insect groups with NMDS with four different method approaches; Table S4: Statistical analysis of bacterial community structure between edible insect groups with PCoA with four different method approaches; Table S5: Statistical analysis of relative abundance of selected bacterial families of food quality and safety significance in edible insect groups.
