# Supplementary material for: Comprehensive Analysis of Bacterial Communities and Microbiological Quality of Frozen Edible Insects
Source: Foods. 2025 Jul 1;14(13):2347. doi: 10.3390/foods14132347 (PMC12249108; doi:10.3390/foods14132347)
Supplement: Supplementary file 1 [file foods-14-02347-s001.zip › foods-3705279-supplementary.pdf]

Supplementary Data

Comprehensive Analysis of Bacterial Communities and Microbiological Quality of Frozen Edible Insects

Table S1. Data of raw sequence and sequences obtained after DADA2 was used for denoising, filtering, merging, and chimera removal.

Sample ID	Sample name	Subgroup	raw	trimmed	filtered	denoisedF	denoisedR	merged	nonchim	perc
16S24328	HC1	Adult stage	113961	113956	101349	100691	100541	98647	95582	83.87255
16S24329	HC2	Adult stage	125062	125030	109093	108546	108178	105978	101185	80.90787
16S24330	HC3	Adult stage	111241	111196	95291	94674	94274	91846	83251	74.83841
16S24331	HC4	Adult stage	95035	95004	83729	83383	83117	82020	79614	83.77335
16S24332	HC5	Adult stage	115157	115139	99828	99243	98667	95696	84506	73.38329
16S24333	HC6	Adult stage	122551	122535	104771	104314	104043	102055	96174	78.47672
16S24334	MC1	Adult stage	123179	123159	105893	105216	105012	102421	95063	77.17468
16S24335	MC2	Adult stage	97814	97804	87628	86986	86835	84334	83028	84.88355
16S24336	MC3	Adult stage	110094	110074	97093	96618	96423	94815	93047	84.51596
16S24337	MC4	Adult stage	116496	116464	100627	99234	99018	93981	91311	78.38123
16S24338	MC5	Adult stage	122616	122613	108341	107733	107371	106020	102909	83.92787
16S24339	MC6	Adult stage	101684	101647	90786	89420	89072	84387	79079	77.76936
16S24340	PW1	Juvenile stage	102053	102051	89968	89657	89364	88733	82969	81.29991
16S24341	PW2	Juvenile stage	108309	108302	96365	96070	95681	95185	94704	87.43872
16S24342	PW3	Juvenile stage	127355	127351	113066	112764	112061	111350	110496	86.76220
16S24343	PW4	Juvenile stage	92966	92962	83214	83061	82747	82389	82006	88.21074
16S24344	PW5	Juvenile stage	95934	95926	83975	83690	83458	82936	82070	85.54840
16S24345	PW6	Juvenile stage	118729	118728	104703	104391	103921	103075	101576	85.55281
16S24346	SM1	Juvenile stage	82837	82832	71139	70732	69940	68966	67732	81.76539
16S24347	SM2	Juvenile stage	102828	102825	90268	89554	89617	88525	86424	84.04715
16S24348	SM3	Juvenile stage	102221	102217	87793	87247	87083	86025	84687	82.84697
16S24349	SM4	Juvenile stage	97434	97397	85495	85141	85059	84427	83714	85.91867
16S24350	SM5	Juvenile stage	90572	90570	80138	79836	79679	79075	78221	86.36334
16S24351	SM6	Juvenile stage	103306	103299	91620	90890	90642	89285	87233	84.44137
16S24352	BW1	Juvenile stage	106764	106761	93974	93396	93321	92465	88041	82.46319
16S24353	BW2	Juvenile stage	124098	124086	112264	111695	111336	110578	103257	83.20601
16S24354	BW3	Juvenile stage	121021	121013	106521	105957	105790	104865	101385	83.77472
16S24355	BW4	Juvenile stage	92935	92934	81848	81466	81331	80786	77780	83.69290
16S24356	BW5	Juvenile stage	112220	112206	98861	98267	98012	96454	86204	76.81697
16S24357	BW6	Juvenile stage	111982	111979	98181	97710	97473	96665	89509	79.93160
		**Average**	108281.8	108268.667	95127.4	94586.0667	94302.2	92799.4667	89091.9	82.39953

Note: Abbreviations: BW: bamboo worms, HC: house crickets, MC: mole crickets, PW: palm weevils, and SM: Silkworms (silk moth larvae).

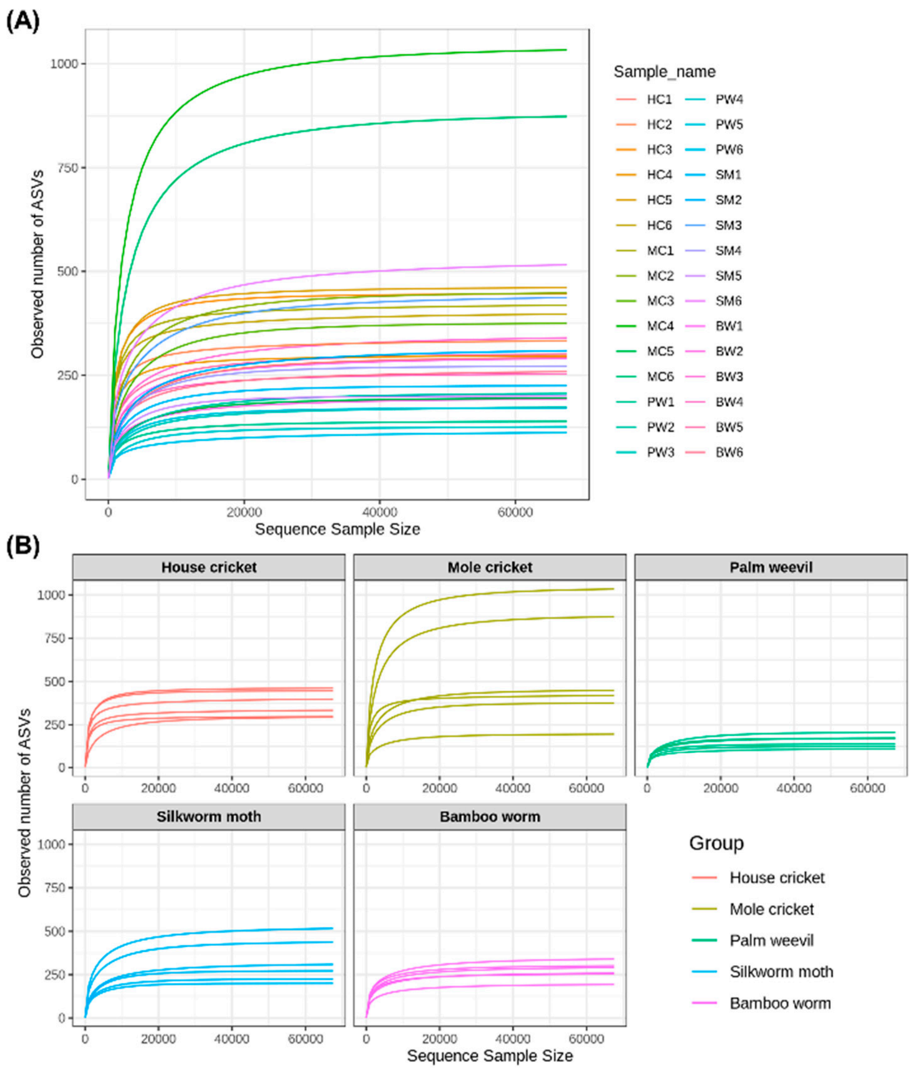

Figure S1. Rarefaction curves of observed microbial ASVs detected in five frozen edible insect groups. (A) Overall observed numbers in all samples. (B) Observed numbers in individual samples of each insect group.

Table S2. Statistical analysis of bacterial community structure among edible insect groups.

	Distance method	Stress	ADONIS			ANOSIM	
			R^2	pseudo F	*p*-value	R Statistic	*p*-value*
NMDS	UniFrac1 (Unweighted UniFrac)	0.0808	0.4553	5.2235	0.001	0.8477	0.001
	UniFrac2 (Weighted UniFrac)	0.0977	0.5621	8.0239	0.001	0.6155	0.001
	Bray-Curtis	0.1252	0.4549	5.2156	0.001	0.7492	0.001
	Jaccard	0.1252	0.3464	3.3129	0.001	0.7492	0.001
PCoA	UniFrac1 (Unweighted UniFrac)	-	0.4552	5.2219	0.001	0.8510	0.001
	UniFrac2 (Weighted UniFrac)	-	0.5443	7.4650	0.001	0.6214	0.001
	Bray-Curtis	-	0.4778	5.7176	0.001	0.7458	0.001
	Jaccard	-	0.3672	3.6269	0.001	0.7458	0.001

* *p*-values given from ANOSIM method are adjusted by Bonferroni method.

Table S3. Statistical analysis of bacterial community structure between frozen edible insect groups with NMDS with four different method approaches.

Distance metrics	Group vs Group	PERMANOVA			ANOSIM	
		R^2	pseudo F	*p*-value	R Statistic	*p*-value*
Bray-Curtis distance	HC vs MC	0.234	3.050	0.050	0.421	0.33
	HC vs PW	0.370	5.866	0.003	0.680	0.06
	HC vs SM	0.374	5.965	0.004	0.691	0.11
	HC vs BW	0.462	8.592	0.004	0.667	0.14
	MC vs PW	0.281	3.907	0.003	0.824	0.01
	MC vs SM	0.258	3.477	0.005	0.733	0.06
	MC vs BW	0.353	5.462	0.006	0.743	0.03
	PW vs SM	0.318	4.654	0.003	1.000	0.03
	PW vs BW	0.406	6.848	0.001	0.994	0.04
	SM vs BW	0.402	6.728	0.002	1.000	0.03
Unweighted UniFrac	HC vs MC	0.210	2.657	0.033	0.426	0.29
	HC vs PW	0.394	6.498	0.004	0.993	0.06
	HC vs SM	0.400	6.677	0.005	0.969	0.05
	HC vs BW	0.448	8.108	0.005	0.954	0.02
	MC vs PW	0.309	4.474	0.004	0.956	0.02
	MC vs SM	0.296	4.214	0.002	0.937	0.02
	MC vs BW	0.355	5.499	0.003	0.909	0.01
	PW vs SM	0.317	4.647	0.001	1.000	0.02
	PW vs BW	0.332	4.967	0.002	0.980	0.02
	SM vs BW	0.372	5.919	0.001	1.000	0.06
Weighted UniFrac	HC vs MC	0.369	5.857	0.013	0.537	0.09
	HC vs PW	0.533	11.411	0.006	0.806	0.04
	HC vs SM	0.601	15.049	0.002	0.800	0.03
	HC vs BW	0.623	16.493	0.001	0.776	0.04
	MC vs PW	0.301	4.301	0.005	0.587	0.03
	MC vs SM	0.291	4.112	0.005	0.487	0.02
	MC vs BW	0.393	6.478	0.005	0.648	0.03
	PW vs SM	0.267	3.650	0.016	0.431	0.06
	PW vs BW	0.372	5.927	0.003	0.583	0.07
	SM vs BW	0.442	7.935	0.004	0.741	0.04
Jaccard	HC vs MC	0.180	2.194	0.076	0.421	0.29
	HC vs PW	0.271	3.711	0.005	0.680	0.09
	HC vs SM	0.271	3.721	0.002	0.691	0.13
	HC vs BW	0.338	5.114	0.004	0.667	0.11
	MC vs PW	0.203	2.542	0.004	0.824	0.02
	MC vs SM	0.189	2.330	0.005	0.733	0.02
	MC vs BW	0.259	3.491	0.007	0.743	0.01
	PW vs SM	0.223	2.863	0.002	1.000	0.04
	PW vs BW	0.287	4.016	0.001	0.994	0.03
	SM vs BW	0.282	3.936	0.005	1.000	0.05

Note: Abbreviations: BW: bamboo worms, HC: house crickets, MC: mole crickets, PW: palm weevil larvae, and SM: silkworms (silk moth larvae). * *p*-values given from ANOSIM method are adjusted by Bonferroni method.

Table S4. Statistical analysis of bacterial community structure between edible insect groups with PCoA with four different method approaches.

Distance metrics	Group vs Group	PERMANOVA			ANOSIM	
		R^2	pseudo F	*p*-value	R Statistic	*p*-value*
Bray-Curtis distance	HC vs MC	0.224	2.887	0.010	0.459	0.260
	HC vs PW	0.378	6.065	0.002	0.807	0.050
	HC vs SM	0.404	6.783	0.003	0.767	0.030
	HC vs BW	0.439	7.828	0.002	0.867	0.010
	MC vs PW	0.279	3.866	0.001	0.693	0.030
	MC vs SM	0.247	3.288	0.005	0.481	0.010
	MC vs BW	0.360	5.613	0.007	0.843	0.010
	PW vs SM	0.359	5.608	0.005	0.735	0.020
	PW vs BW	0.428	7.486	0.004	0.880	0.050
	SM vs BW	0.531	11.309	0.004	1.000	0.020
Unweighted UniFrac	HC vs MC	0.210	2.655	0.036	0.422	0.320
	HC vs PW	0.394	6.510	0.007	0.993	0.020
	HC vs SM	0.401	6.691	0.003	0.974	0.040
	HC vs BW	0.448	8.119	0.004	0.957	0.040
	MC vs PW	0.309	4.480	0.002	0.956	0.040
	MC vs SM	0.297	4.224	0.003	0.943	0.030
	MC vs BW	0.355	5.506	0.006	0.915	0.030
	PW vs SM	0.317	4.637	0.004	1.000	0.060
	PW vs BW	0.331	4.951	0.001	0.980	0.030
	SM vs BW	0.371	5.898	0.005	1.000	0.020
Weighted UniFrac	HC vs MC	0.338	5.111	0.011	0.493	0.140
	HC vs PW	0.507	10.299	0.003	0.819	0.040
	HC vs SM	0.589	14.303	0.001	0.826	0.040
	HC vs BW	0.612	15.772	0.003	0.750	0.060
	MC vs PW	0.303	4.356	0.003	0.624	0.010
	MC vs SM	0.301	4.316	0.004	0.537	0.020
	MC vs BW	0.396	6.544	0.002	0.678	0.020
	PW vs SM	0.278	3.857	0.014	0.454	0.120
	PW vs BW	0.372	5.922	0.002	0.593	0.040
	SM vs BW	0.435	7.691	0.004	0.730	0.010
Jaccard	HC vs MC	0.172	2.072	0.019	0.459	0.270
	HC vs PW	0.273	3.763	0.003	0.807	0.070
	HC vs SM	0.295	4.175	0.003	0.767	0.020
	HC vs BW	0.319	4.678	0.002	0.867	0.020
	MC vs PW	0.207	2.608	0.002	0.693	0.030
	MC vs SM	0.194	2.413	0.004	0.481	0.030
	MC vs BW	0.265	3.612	0.001	0.843	0.030
	PW vs SM	0.260	3.518	0.004	0.735	0.030
	PW vs BW	0.309	4.462	0.003	0.880	0.020
	SM vs BW	0.377	6.042	0.002	1.000	0.050

Note: Abbreviations: BW: bamboo worms, HC: house crickets, MC: mole crickets, PW: palm weevils, and SM: Silkworms (silk moth larvae). * *p*-values given from ANOSIM method are adjusted by Bonferroni method.

Table S5. Statistical analysis of relative abundance of selected bacterial families of food quality and safety significance in edible insect groups.

	Relative abundance (%)					
**Insect group**	***Bacillaceae***	***Enterobacteriaceae***	***Lactobacillaceae***	***Listeriaceae***	***Staphylococcaceae***	***Streptococcaceae***
BW	0.01 ± 0.02^{a}	17.20 ± 4.89^{ab}	16.90 ± 11.5^{b}	2.26 ± 8.51^{a}	0.05 ± 0.03^{a}	31.20 ± 17.00^{b}
HC	0.00 ± 0.00^{a}	7.30 ± 3.47^{a}	0.01 ± 0.01^{a}	0.00 ± 0.01^{a}	0.03 ± 0.13^{a}	0.76 ± 0.66^{a}
MC	0.28 ± 0.36^{ab}	11.80 ± 13.60^{ab}	0.69 ± 1.77^{ab}	1.30 ± 2.49^{a}	8.85 ± 7.17^{b}	3.60 ± 8.26^{ab}
PW	0.04 ± 1.22^{ab}	28.60 ± 21.90^{ab}	2.28 ± 2.23^{ab}	0.00 ± 0.00^{a}	0.04 ± 0.07^{a}	18.40 ± 23.40^{b}
SM	2.30 ± 2.24^{b}	26.70 ± 17.60^{b}	0.15 ± 0.22^{a}	0.00 ± 0.02^{a}	1.97 ± 2.91^{ab}	29.40 ± 20.70^{b}

Note: Abbreviations: BW: bamboo worms, HC: house crickets, MC: mole crickets, PW: palm weevils, and SM: silkworms (silk moth larvae); Values given as means relative abundance ± SD; different letters across columns indicate statistically significant differences ($p < 0.05$) in percentages of relative abundance among the insect groups (Kruskal-Wallis and Dunn’s tests).
